# SOCIAL SUPPORT–HEALTH LINKAGES DURING A MAJOR LIFE EVENT: A NEW SPIN ON STRESS-BUFFERING EFFECTS

**DOI:** 10.1093/geroni/igad104.1916

**Published:** 2023-12-21

**Authors:** Gloria Luong

**Affiliations:** Colorado State University, Fort Collins, Colorado, United States

## Abstract

Supportive social relationships are posited to confer benefits to longer-term health and well-being, and may be especially protective in the face of adversity, when people can draw on these resources to cope with stressful events (stress buffering effects). Yet, most previous studies have used retrospective, cross-sectional designs, limiting the ability to delineate how the linkages between social support and health may change prospectively as people anticipate and prepare for an upcoming stressor, such as relocating into a senior housing facility and adjusting to the experiences afterward. The Relocation and Transitional Experiences (RELATE) study tracked 154 older adults using a prospective longitudinal measurement burst design as they transitioned into a senior housing facility. The waves (bursts) of assessments were based on the following schedule: Burst 1 (2 weeks prior to the move), Burst 2 (2 weeks after the move), Burst 3 (1.5 months after the move), and Burst 4 (~3-6 months after the move). At each burst, participants completed questionnaires on their health and well-being, social relationships and social support, and relationship satisfaction. We examined changes in the strength of social support-health linkages across bursts. Results show that social-support health linkages were weakest and least consistent during the early stages of the relocation stressor (Burst 2 and 3) but that by Burst 4, support-health linkages were mostly restored similar to pre-move levels (Burst 1). These findings suggest that stress buffering effects need to be understood within the context of how stressful experiences unfold over time for older adults.

